# Optic Chiasm Glioma in an Older Adult Patient

**DOI:** 10.7759/cureus.51614

**Published:** 2024-01-03

**Authors:** Ulviyya Gasimova, Osasu Adah, Matahi Muradova, Kaleigh Roberts, Sonika Dahiya, Rashmi Verma, Lokesh Rukmangadachar

**Affiliations:** 1 Neurology, SSM (Sisters of St. Mary) Health Saint Louis University School of Medicine, St. Louis, USA; 2 Ophthalmology, SSM (Sisters of St. Mary) Health Saint Louis University School of Medicine, St. Louis, USA; 3 Ophthalmology, National Center Ophthalmology named after Academician Zarifa Aliyeva, Baku, AZE; 4 Pathology and Laboratory Medicine, Washington University School of Medicine, St. Louis, USA; 5 Ophthalmology, Northwell Health, New York, USA; 6 Neurology, BSW (Baylor Scott & White) Health, Austin, USA

**Keywords:** persistent contrast enhancement, demyelinating disease mimickers, glioblastoma, glioma, optic chiasm

## Abstract

We present a case of an adult patient experiencing progressive visual loss. An initial presentation was concerning for neuromyelitis optica with optic chiasm involvement. However, persistent contrast enhancement observed in follow-up brain and orbit images raised suspicion for optic tract malignant neoplasm. Histopathological evolution of optic nerve biopsy confirmed the diagnosis of an optic chiasm glioma. The patient was then referred to oncology for chemotherapy.

## Introduction

Optic pathway primary tumors are uncommon in adults, with a prevalence of nearly 5% of brain tumors in the pediatric population. They can be divided into glioma, meningioma, ganglioglioma, and primary lymphoma [1]. Gliomas can affect all the segments of the central nervous system including the optic pathway. The optic chiasm is the most affected segment. Gliomas can be sporadic and associated with neurofibromatosis type 1 (NF1) and other genetic conditions, having better visual prognosis in the latter cases [2].

Our case presents a progressive visual disturbance initially thought to be a demyelinating condition; however, further workup with histopathological evaluation confirms the diagnosis of an optic chiasm glioma.

## Case presentation

**Figure 1:**
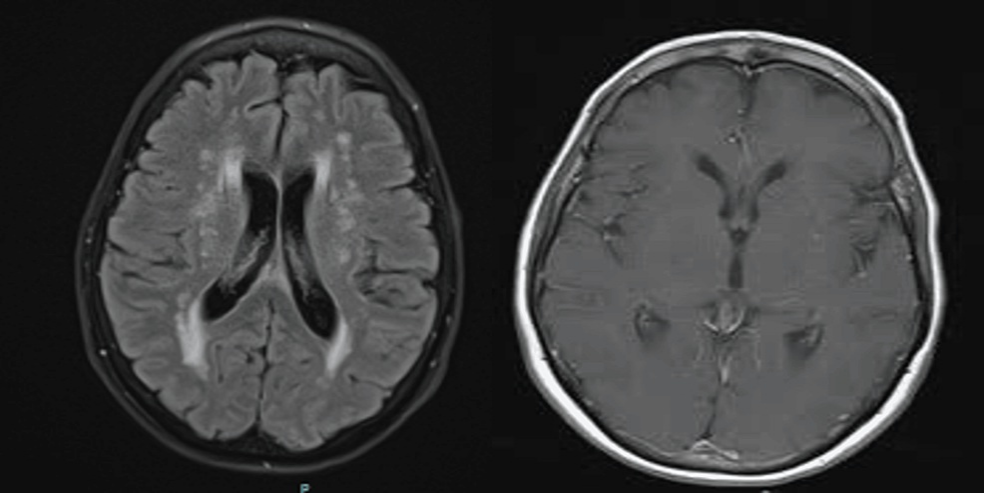
MRI brain wwo contrast. FLAIR (left) reveals periventricular white matter hyperintensities, and T1 with contrast (right) shows no contrast enhancement. wwo: With and without; FLAIR: Fluid-attenuated inversion recovery.

**Figure 2:**
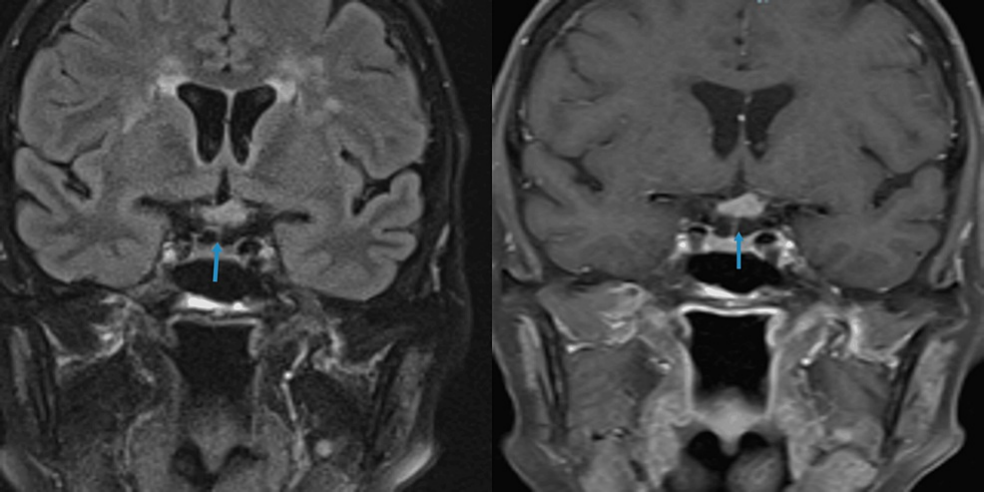
MRI orbits wwo contrast. T2 FLAIR (left) reveals signal hyperintensity and T1 with contrast in the coronal section (right) shows homogeneous enhancement of the left retro-orbital optic tract. wwo: With and without; FLAIR: Fluid-attenuated inversion recovery.

A woman in her 70s with a past medical history remarkable for hypertension controlled by diet presented to the ophthalmology clinic for cataract evaluation due to a steady slow decline in vision in both eyes. Visual acuity during this visit in her right and left eye was 20/25 and 20/30, respectively, with no afferent pupillary defect (APD). Otherwise, she had a healthy appearance of optic nerves and retinal vessels in both eyes. The patient presented four months later due to worsening blurred vision in the left eye. Vision in her right and left eye were 20/30 and 20/40, respectively, with no APD. During this period, she denied any other neurological concerns. Anterior and posterior exams revealed similar findings as before. Ocular coherence testing (OCT) of the macula showed a normal retina cross-section of both eyes. At this time, the patient was recommended to proceed with cataract surgery, starting with the left eye. Due to the progressively worsening visual disturbances of the left eye, the patient presented about three weeks later to the ophthalmology clinic. Vision in the right and left eyes were 20/30 and 20/200, respectively. There was a positive APD in the left eye. Confrontation visual fields (CVF) of the left eye showed partial temporal and nasal defects. A posterior exam of the left eye showed possible temporal pallor of the nerve. The repeat OCT of the macula was unremarkable. OCT of the nerve showed mild retinal nerve fiber layer (RNFL) thinning of the left eye compared to the right eye. Computer tomography (CT) of the chest, MRI of cervical and thoracic spines, and positron emission tomography (PET) of the brain and body, which was obtained to rule out primary tumors, were all unremarkable. MRI of the brain and orbits with and without (wwo) contrast was obtained, which showed T2/fluid-attenuated inversion recovery (FLAIR) signal hyperintensity and homogeneous enhancement of the left retro-orbital optic tract proximally (Figures 1, 2). Serum and cerebrospinal fluid (CSF) studies for inflammatory and infectious sources, aquaporin 4, and anti-myelin oligodendrocyte glycoprotein (anti-MOG) antibodies were unremarkable.

**Figure 3:**
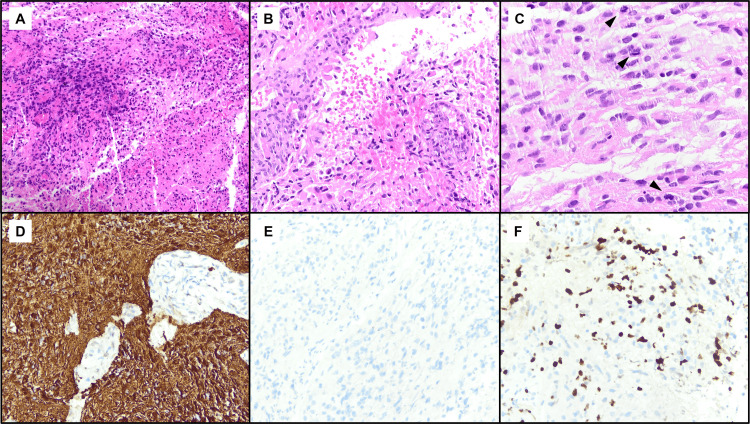
(A-C) Hematoxylin and eosin stain. (D) Glial fibrillary acidic protein stain. (E) Isocitrate dehydrogenase (IDH1, R132H). (F) Ki-67.

The patient was started on intravenous (IV) solumedrol 1 gram a day for a total of five days. Plasmapheresis (PLEX) was then initiated for a total of five days. Three days into the hospital stay, the patient started to complain of visual changes in her right eye. With concern for malignant disease in lieu of persistent contrast enhancement of an optic chiasm lesion on follow-up images, it was decided to proceed with a nerve biopsy. Her left optic nerve was biopsied, which confirmed the diagnosis of optic pathway glioma (Figure 3). It was identified to be a high-grade glioma with positive glial fibrillary acidic protein (GFAP) and negative mutant IDH-1. The patient was referred for radiation therapy and was started on oral chemotherapy with temozolomide.

## Discussion

As mentioned above, optic pathway primary tumors are rare to see in clinical practice. The tumor affecting the optic chiasm is seen in less than 20% of cases. In 80% of patients, there is an initial phase of visual deterioration followed by stabilization [1].

Children under 10 years are most commonly affected by optic pathway gliomas, constituting 3%-5% of all central nervous system (CNS) tumors in this population. It is noteworthy that optic pathway gliomas are also seen in the elderly population, making the age range between birth and late 70s. Optic pathway gliomas equally affect men and women; however, in cases reported to date, male predominance was noted [3,4]. Huang et al. described that the mean age at presentation is 8.8 years, with 71% of cases found in the first decade of life and 90% within the first two decades [1]. Although these tumors are typically regarded as slow-growing, the pace of progression can be variable. Over time, 85% will lose some vision, with 25% retaining vision between 20/20 to 20/40. About 60% will develop vision worse than 20/300. Proptosis is seen in 95% of patients with glioma and is the most common presenting sign.

Malignant optic nerve glioma is an aggressive and rare type, which was first described in 1973 [5]. This condition tends to affect old adults and patients present with rapid loss of vision, usually misdiagnosed as anterior ischemic optic neuropathy or optic neuritis. All patients tend to progress to complete vision loss within a month of presenting [5]. In 2004, Wabbels et al. reported 45 cases of malignant optic glioma [6]. In 2015, Traber et al. described five more patients with the same nosology [7]. In most of these cases, the diagnosis was made based on MRI findings being consistent with T2 FLAIR hyperintensity and T1 contrast enhancement. Initial diagnoses in these presented cases were thought to be optic neuritis, brain tumor to optic neuritis, or neurosarcoidosis. Only during further workup based on imaging findings, suspicion for optic pathway gliomas was raised [6,7].

In 2022, Merchancano-Esquivel et al. reported a case of a 77-year-old man with bilateral progressive vision loss, initially thought to be non-arteritic anterior ischemic optic neuropathy. Further workup revealed a glioma of the optic nerve in settings of non-uniform contrast enhancement of the optic chiasm and left optic tract on MRI. The diagnosis was confirmed by biopsy results [8]. In the reported cases, male patients were predominant. As discussed, the diagnosis of optic pathway gliomas is made based on specific imaging findings and is confirmed by biopsy results. Treatment includes surgery, radiation, and chemotherapy, with vincristine being the first chemotherapist option [9].

## Conclusions

It is important to always keep in mind red flags for each suspected pathology and to initiate further workup if suspicion for another cause is high. In our case, persistent contrast enhancement in follow-up brain and orbit images involving optic chiasm raised a suspicion for malignancy, which prompted an optic nerve biopsy, confirming a diagnosis of optic chiasm glioma. A timely approach helped us to navigate the management with the hematology-oncology team.

It is crucial to increase awareness toward distinguishing between demyelinating and other more rare pathologies affecting the optic system and causing visual disturbances. If these cases are not approached from a wide perspective, this can be a real challenge. Timely suspicion for another underlying pathology is essential to modify the workup accordingly as the management can be crucially different. Published data will help to provide better care for patients with optic tract gliomas.
